# Paradoxical effect of dopamine-agonists on IGF-1 in patients with prolactinoma: the role of weight

**DOI:** 10.1186/s12902-024-01622-4

**Published:** 2024-06-20

**Authors:** S. Caprio, T. Pilli, S. Cantara, F. Sestini, C. Fioravanti, C. Ciuoli, C. Dalmiglio, A. Corbo, M. G. Castagna

**Affiliations:** 1 Section of Endocrinology, Department of Medical, Surgical and Neurogical Sciences, University of Siena, Siena, Italy; 2 Laboratory of Clinical and Translational Research, University of Siena, Siena, Italy

**Keywords:** Prolactinoma, Dopamine-agonists, IGF-1, Body mass index

## Abstract

**Purpose:**

An increase of IGF-1 has been reported during therapy with dopamine agonists (DA) for prolactinomas; in such cases a correct diagnosis is pivotal to avoid an unnecessary reduction or withdrawal of DA, which are needed to maintain normal prolactin levels. This study was aimed to measure IGF-1 levels, at baseline and during follow-up, in a cohort of patients with prolactinoma, treated with cabergoline, stratified by body mass index.

**Methods:**

We retrospectively enrolled 35 patients (15 F/20 M; age m ± SD, years: 43.4 ± 13.7) with prolactinoma (21 microadenomas and 14 macroadenomas) who were followed-up at the Endocrinology Unit, in Siena, and with available pituitary hormone assessment at baseline and during follow-up (m ± SD, years: 2.74 ± 0.55).

**Results:**

IGF-1 increased in the whole cohort, but remaining within normal range, except two patients, in whom acromegaly was ruled out with oral glucose tolerance test. After dividing patients by weight, this trend was confirmed only in subjects with overweight and obesity (OV/OB) (*p* = 0.04). Interestingly, the reduction of prolactin levels was significantly greater in the OV/OB compared to normal-weight patients (median decrease of 97.5% versus 88.2%, *p* = 0.04).

**Conclusions:**

Since DA and normalization of prolactin are known to improve insulin sensitivity, we speculated they have favored the increase of IGF-1 in OV/OB. Our results should be confirmed and the hypothesis proven by further studies.

## Introduction

Prolactin (PRL) and growth hormone (GH) can be co-secreted by mammosomatotroph cells, that account for up to 50% of pituitary secreting cells. This co-secretion can be present ab initio (mixed adenomas) or develop later, with hyperprolactinemia preceding the diagnosis of acromegaly. The prevalence of this phenomenon ranges between 4 and 10% in patients with prolactinoma [1]. Dopamine-agonists (DA) represent the first line treatment in patients with hyperprolactinemia; however, they can be also used in acromegaly, As a result, patients with acromegaly may be misdiagnosed or receive a late diagnosis, although the prevalence of silent acromegaly seems to be very low [2] On the other hand it has been reported that DA can cause an increase of the IGF-1 levels [3]. Several hypotheses have been formulated to explain this paradoxical effect but none of them are conclusive. A reduction in glucose tolerance and a raise in fasting insulin have been found in patients with hyperprolactinemia [4]. A U-shaped association between IGF-I serum concentrations and increased HOMA values has been reported; namely deviations of IGF-I regulation in both directions are related to insulin resistance [5]. Therefore, the link between prolactin normalization and IGF-1 increase may be represented by the amelioration of insulin sensitivity as a result of direct or indirect effect of DA. The aim of this study was to evaluate IGF-1 levels in a cohort of patients with prolactinoma at baseline and during follow-up stratified by body mass index (BMI).

## Subjects and methods

We retrospectively enrolled 35 patients (15 F/20 M; age m ± SD, years: 43.4 ± 13.7, range 20–78) with prolactinoma (21 microadenomas and 14 macroadenomas), treated with cabergoline, who were followed-up at the Endocrinology Unit (University of Siena) from 2006 to 2021 (m ± SD, years: 2.74 ± 0.55, range 2–4) and with available pituitay hormone assessment at baseline and during follow-up. Prolactinomas were diagnosed based on symptoms, either menstrual abnormalities and/or galactorrhea in females and erectile dysfunction in males, and the presence of a pituitary adenoma, after ruling out other causes of hyperprolactinemia. According to BMI patients were classified as normal-weight (BMI ≤ 24.9; *n* = 10) or overweight/obese (BMI ≥ 25; *n* = 25). Only one patient (2.8%) was affected by diabetes mellitus. Patients with a previous diagnosis of acromegaly or with reduced IGF-1 levels at baseline were excluded from the study. IGF-1 levels was determined at baseline and at 12, 18, 24 and 36 months during follow-up. IGF-1 (IMMULITE® 2000) and PRL (Beckman Coulter Access 2) were assessed by chemiluminescence and for IGF-1 age-specific reference intervals were used. This study was approved by the Ethical Committee of the University Hospital of Siena and patients gave a written informed consent before participating.

### Statistics

Epidemiological data are shown as mean value ± standard deviation, for parametric data, or as median ± interquartile range for nonparametric data. Nominal variables were analyzed using χ2 Test (or Fisher Test when appropriate) and continuous variables using unpaired Student t-test or Mann Whitney U test depending on the normality of the variables. Friedman Test was used to analyze PRL and IGF-1 changes during follow-up. Since IGF-1 levels vary depending on age, they were normalized by age. StatView for Windows (version 5.0.1, SAS Institute, Cary, NC) was used for the statical analysis. *P*-values < 0.05 were considered statistically significant.

## Results

Clinical and hormomal parameters of the whole cohort and according to BMI are summarized in Table 1.

**Table 1:** Clinical and hormonal parameters of the study population

	Whole Cohort (*n* = 35)			Normal weight (*n* = 10)			Overweight/Ob*ese (n* = 25)			p*
	Baseline	12–18 months	Last visit	Baseline	12–18 months	Last visit	Baseline	12–18 months	Last visit	
**Sex** (F/M)	15/20			6/4			9/16			0.19
**Age at diagnosis** (years) mean ± SD	43.4 ± 13.7			38.4 ± 15.3			45.4 ± 12.8			0.13
**Type of adenoma** micro/macro	21/14			6/4			15/10			ns
**Weight** (kg) median ± IQR	77.42 ± 24.62	82.0 ± 31.5	79.0 ± 25.75	61.5 ± 15.0	62.5 ± 8.0	66.0 ± 14.75	87.0 ± 36.55	94.0 ± 25.5	89.5 ± 20.0	0.0
**BMI** (kg/m2) median ± IQR	28.0 ± 9.0	30.0 ± 9.0	28.0 ± 8.0	21.5 ± 5.0	23.0 ± 3.0	22.0 ± 6.0	30.0 ± 6.87	31.0 ± 7.5	30.0 ± 7.5	0.0
**Cabergoline dose** (mg) median ± IQR		1.0 ± 1.5	1.0 ± 1.5		1.0 ± 0,87	1.0 ± 1.94		1.0 ± 1.5	0.87 ± 1.5	ns
**Prolactin (**ng/ml) median ± IQR	137.7 ± 357.4	7.6 ± 9.6	6.5 ± 8.2	110.2 ± 93.0	11.2 ± 18.8	6.7 ± 10.9	199.0 ± 559.4	6.6 ± 7.3	6.3 ± 8.3	ns
**GH (**ng/ml) median ± IQR	0.10 ± 0.52	0.21 ± 0.89	0.14 ± 0.53	0.19 ± 0.56	0.39 ± 2.64	0.50 ± 1.83	0.08 ± 0.47	0.19 ± 0.70	0.09 ± 0.20	ns
**IGF-1 (**ng/ml) median ± IQR	144.0 ± 91.0	175.5 ± 85.0	158.0 ± 54.5	164.5 ± 139.0	181.5 ± 83.6	170.0 ± 48.7	132.0 ± 50.0	172.5 ± 88.0	156.0 ± 69.0	0.04#
**FT4 (**pg/ml) median ± IQR	7.4 ± 1.8	7.7 ± 1.4	8.1 ± 0.9	8.0 ± 1.8	8.0 ± 1.3	7.6 ± 0.9	7.3 ± 1.8	7.5 ± 1.5	8.2 ± 0.8	ns
**Testosterone (**pg/ml) median ± IQR	1.38 ± 1.92	2.91 ± 1.07	3.20 ± 1.58	2.05 ± 3.39	2.77 ± 0.28	3.53 ± 1.73	1.38 ± 1.90	3.00 ± 1.44	3.20 ± 1.71	ns

χ2 Test was used for sex and type of adenoma, unpaired Student t-test for age and Mann Whitney U test for the remaining parameter analysis

A *p* < 0.05 is considered significant

^*^The *p* is the result of comparison between normal weight versus overweight/obese at each time point

^#^The *p* is the result of comparison between normal weight versus overweight/obese at baseline

### PRL and IGF-1 levels at diagnosis and during follow-up

PRL levels at diagnosis ranged between 40.3 ng/ml and 3264 ng/ml (median ± IQR 138.7 ± 357.4). PRL was significantly lower in female patients (median ± IQR 60.9 ± 75.4 ng/ml versus 354.4 ± 955.4 ng/ml in males, *p* = 0.0003) and in microadenomas (median ± IQR 69.9 ± 88.0 ng/ml versus 679.0 ± 965.8 ng/ml in macroadenomas, *p* =  < 0.0001). No significant differences were observed among weight categories (*p* = 0.29). Sex and tumor size did not affect IGF-1 values at baseline while IGF-1 was significantly lower in overweight or obese than in normal-weight patients (median ± IQR 164.5 ± 139.0 ng/ml versus 132.0 ± 50.0 ng/ml, *p* = 0.04).

**Fig. 1:**
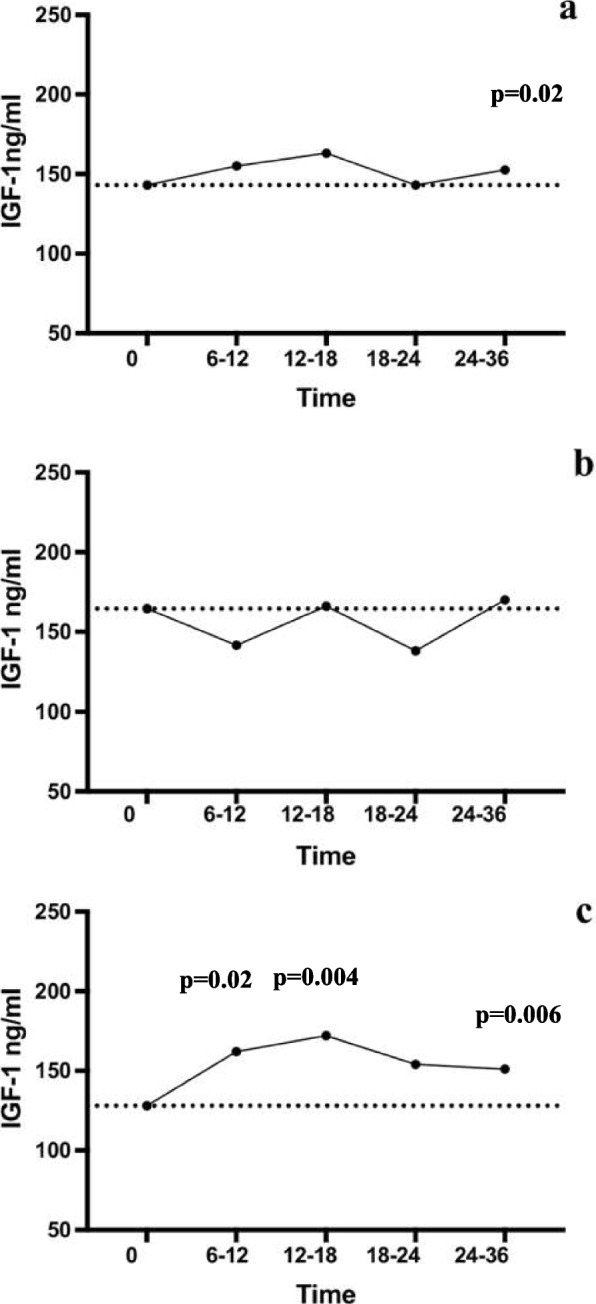
IGF-1, expressed as median at different time points in the whole cohort (**a**), normal-weight (**b**) and overweight and obese (**c**) patients. Friedman Test was used to compare the repeated measurements of IGF-1 over time upon cabergoline therapy. A *p* < 0.05 was considered significant

IGF-1 values showed a significant increase, during DA therapy, in the whole cohort (*p* = 0.02; Fig. 1a) and, when body weight was taken into account, this trend was confirmed only in patients with overweight and obesity (*p* = 0.04; Fig. 1c), while in normal weight patients IGF-1 did not significantly change (Fig. 1b). IGF-1 increased since the early follow-up but remaining within normal range with the exception of two cases, with a percentage of the upper limit of normal for the subject age (%ULN) of 142% and 166% respectively, who underwent oral glucose tolerance test (OGTT) which ruled out acromegaly.

PRL values significantly decreased with DA therapy (*p* < 0.0001). Interestingly the reduction of prolactin levels was significantly higher in the overweight/obese patients compared to those with normal weight (median decrease of 97.5% versus 88.2%, *p* = 0.04) but there was no difference of cabegorgoline dose between the two groups at any time. Moreover there was no correlation between cabergoline dose and IGF-1 change.

### FT4, estrogens and testosterone levels at baseline and during follow-up

FT4 were measured at baseline and during follow-up and no significant changes were observed neither in the whole cohort nor after dividing patients based on the BMI. None of the female patients, in the fertile age, was on estrogen therapy and menstrual cycle abnormalities were corrected by the prolactin normalization. Testosterone levels were reduced in 16 out of 20 male patients, as expected in presence of hyperprolactinemia, and normalized following prolactin decrease induced by cabergoline. There was no difference in terms of hormone levels, during follow-up, between patients with normal weight and those with overweight or obesity.

## Discussion

An increase of IGF-1 has been reported during therapy with dopamine agonists for prolactinomas; in such cases a correct diagnosis is pivotal to avoid an unnecessary reduction or withdrawal of DA, which are needed to maintain normal prolactin levels. In our study we observed a significant change of IGF-1 levels during follow-up in the whole cohort. These results are consistent with those by Akirov et al. [3] and more recently by Bona et al. [2]. Moreover, we found that, after dividing the patients by weight, IGF-1 increased significantly only in subjects with overweight and obesity. Previously, only one study by Andereggen et al. [6] has evaluated the effect of body weight on IGF-1 levels in patients with prolactinomas treated with DA. The authors observed an increase of IGF-1 in 15 out of 20 patients, who had also basal IGF-1 levels lower than patients with stable IGF-1 during follow-up. However, in this study the change of IGF-1 levels, according to the body weight, did not reach statistical significance. The mechanisms underlying IGF-1 increase during DA therapy are still unknown. We speculated that this phenomenon may be the consequence of the effect of DA or prolactin on the insulin sensitivity. Low and high IGF-I levels are both related to insulin resistance with a U-shaped effect [5]. Obesity is commonly linked to insulin resistance and several studies report that IGF-I levels are low in obese patients [7]. Accordingly, we found that basal IGF-1 was significantly lower in patients with overweight and obesity compared to those with normal weight. Also, it has been observed that hyperprolactinemia is associated with a worse glucose tolerance and an increased risk of insulin resistance [8] and that a significant reduction in serum glucose levels occurs in the first 3 months of DA therapy, with a further improvement of glucose tolerance if treatment is continued for at least 6 months [9]. This effect is a consequence of a better insulin sensitivity during DA therapy due to the beneficial effect of prolactin normalization and several mechanisms involving dopaminergic signaling. Interestingly, in our study the increase of GF-1 was observed since the early follow-up. Other causes of IGF-1 increase (weight loss, oral contraceptives or replacement therapy with l-thyroxine or testosterone) were ruled out. Patient weight and BMI did not significantly differ across the study, FT4 was normal at all time points, testosterone was not different between patients with normal weight and those with overweight and obesity and none of female patients, in the fertile age, was on estrogen therapy. By contrast, the reduction of prolactin levels was significantly higher in the overweight/obese patients compared to those with normal weight. Therefore, we hypothesized that the reduction of prolactin by improving insulin sensitivity have favored the increase of IGF-1 in patients with overweight and obesity. However the IGF-1 increase was within normal range with the exception of two cases in whom acromegaly was ruled out.

Our study has some limitations: retrospective and single-center design, relatively small cohort (due to the selection of patients with available clinical and hormonal parameters at baseline and during follow-up), and inability to evaluate insulin resistance. However, our study is the only one, beside Andereggen et al. [4], to have systematically considered the patient weight category while evaluating the effect of DA treatment on IGF-1.

In conclusion, as recommended by the recent guidelines, a measurement of IGF-1 should be done, at diagnosis, in all patients with prolactinomas to rule out a mixed hypersecretion of GH and PRL [10]. While a systematic evaluation of IGF-1 is not recommended during DA treatment unless symptoms or signs suggestive for incipient acromegaly develop [2]. If an increase of IGF-1 is detected, we suggest to monitor if within the normal range, especially in normal weight patients in whom a functional increase of IGF-1 is less frequently observed compared to overweight and obese patients, and to perform OGTT if age-corrected IGF-1 is above the upper limit of the normal range. Our results should be confirmed and the hypothesis proven by further studies.

## Data Availability

All data generated or analysed during this study are included in this published article.
